# Human cerebral organoids and consciousness: a double-edged sword

**DOI:** 10.1007/s40592-020-00116-y

**Published:** 2020-09-07

**Authors:** Andrea Lavazza

**Affiliations:** 1 Centro Universitario Internazionale, Via Garbasso, 42, 52100 Arezzo, Italy; 2 University of Pavia, Pavia, Italy

**Keywords:** Moral status, Personhood, Kant’s humanity formula, Integrated information theory, Chimeras

## Abstract

Human cerebral organoids (HCOs) are three-dimensional in vitro cell cultures that mimic the developmental process and organization of the developing human brain. In just a few years this technique has produced brain models that are already being used to study diseases of the nervous system and to test treatments and drugs. Currently, HCOs consist of tens of millions of cells and have a size of a few millimeters. The greatest limitation to further development is due to their lack of vascularization. However, recent research has shown that human cerebral organoids can manifest the same electrical activity and connections between brain neurons and EEG patterns as those recorded in preterm babies. All this suggests that, in the future, HCOs may manifest an ability to experience basic sensations such as pain, therefore manifesting sentience, or even rudimentary forms of consciousness. This calls for consideration of whether cerebral organoids should be given a moral status and what limitations should be introduced to regulate research. In this article I focus particularly on the study of the emergence and mechanisms of human consciousness, i.e. one of the most complex scientific problems there are, by means of experiments on HCOs. This type of experiment raises relevant ethical issues and, as I will argue, should probably not be considered morally acceptable.

## Introduction

Human consciousness is an elusive object for scientific research. Until a few decades ago it was even doubtful whether it was an entity in the full sense, from the point of view of the ontology of the physical world on which science is based (Searle 1992). Today consciousness has become one of the great unresolved puzzles for scholars, and there is no unanimous consensus on its definition (Gennaro 2018). In accord to a pragmatic definition that still captures a widespread intuition, consciousness is that thing that disappears when we go to sleep at night and reappears when we wake up in the morning. We know that consciousness understood as awareness of the environment and of ourselves is something innate to our very existence, and that it seems to diminish or disappear, for example, in sleep, anesthesia or coma.

What we do not know is how this capacity arises from the activity of our brain (and there are still respectable philosophical theories—heirs to the classical Cartesian position—which suggest that the brain is not enough to explain human consciousness). There are many empirically-based theories that seek an explanation of consciousness in brain activity (I will not mention here purely cognitive theories [cf. van Gulick 2018]), but none seem to be really convincing enough to reach a significant, albeit temporary, consensus in the scientific and philosophical community. It is no coincidence that, just recently, a $20 million contest was launched by the Templeton Foundation to compete for the most accredited (or less discredited) proposals on the origin of consciousness, involving scholars from all around the world (Reardon 2019).

Unraveling the mysteries of consciousness matters not only because the latter is what qualifies us as human beings—i.e. it is what makes sensations have a certain effect on us and seems to characterize our lives in a unique way (Kriegel 2019). It is also important in order to intervene over so-called altered states of consciousness, such as vegetative states, as well as to understand what kind of consciousness certain animals may have and whether a software or a robot can become aware of the environment and their selves in a way similar to us humans (Kahane and Savulescu 2009; Godfrey-Smith 2016).

In this article, the focus will be different from that of research on consciousness in healthy adult human beings. In fact, the rapid development of so-called human brain organoids or human cerebral organoids (HCOs), i.e. brains grown in vitro from stem cells, opens up a completely new scenario worthy of a careful ethical, or better neuroethical (Lavazza 2016), examination in relation to the implications and consequences of neuroscientific research. The fact that in vitro brain organoids can recreate the cytoarchitectonic development typical of a human brain and manifest a complex functional activity, including coordinated electrical activity, suggests that human cerebral organoids, if further developed for medical research and patient care (provided this becomes possible in the near future), may also develop some form of rudimentary consciousness.

It should be noted that this consciousness might initially be simple sentience, i.e. the ability to experience basic sensations, such as thirst or pain, and then become a consciousness that would still not be fully developed compared to that of a healthy adult human being. This hypothesis is based on the assumption that consciousness manifests itself, at least in some features, by degrees instead of immediately appearing in an all-or-nothing form. But even if this were the case, we could not exclude with certainty that a brain grown in vitro, being very similar to a brain grown in utero, might not tend to develop the same functions and capabilities as the latter, except of course for the important differences involved in the ontogenesis process, starting from the lack of interaction with the external environment.

The presence of a degree of consciousness in HCOs would give rise to the ethical issue of what moral status should be attributed to them and, possibly, what rights should come with the attribution of a moral status. The theme I want to consider here is that the development of some form of consciousness in HCOs could also be sought as a goal in itself, in order to unravel the mystery of human consciousness and, consequently, to obtain benefits for all patients with altered states of consciousness who today have no available treatments (cf. Bayne et al. 2019). This type of research, which on the one hand would seem to be of great importance for science and of great utility for medicine, would directly and voluntarily create situations that we would regard as problematic if they occurred as unintended effects of a different type of research. In other words, already today, by trying to create models of human brains for the treatment of diseases, we could end up growing sentient organs, and this would probably raise serious ethical dilemmas.

Now, as mentioned, it cannot be ruled out that some researchers may want to try to develop human brain organoids capable of some form of consciousness in order to study how this phenomenon arises and manifests itself, both to understand its mechanisms and to obtain new treatments to be applied to patients who are currently incurable. Would it be ethically licit to proceed in the way described, whether the desired results are achievable or not? The research on human brain organoids is therefore characterized as a double-edged sword: very promising for both research and personalized care, extremely controversial from an ethical point of view precisely insofar as it could help to achieve results so far unhoped-for, i.e. the understanding of what is one of the biggest questions, if not mysteries, for science.

Based on the current knowledge about human cerebral organoids, I will consider the possibility that they are conscious and what theories of consciousness might fit with the fact that sentient HCOs do not have any relations with the external environment. If a brain organoid could develop some form of awareness, I argue, it should be granted a moral status. That means the HCO would be worth special consideration insofar as it has subjective experiences and is of human origin. In this vein, also the ethical issues concerning chimeras concur to give value and dignity to HCOs grown in a dish. All that considered, the project to study consciousness by experimenting on human brain organoids in the lab should not be considered licit, because it would imply using HCOs as a means and not as an end. And this claim is justified on the basis of avoiding unnecessary pain as a moral imperative in an extended Kantian view.

The article has the following structure. I will briefly explain what human brain organoids are and what their current degree of development is (Sect. 2); I will argue that the evaluation of their possible state of consciousness depends on the theory of consciousness that is adopted and I will discuss the implications of this scientific uncertainty (Sect. 3); I will analyze what it may mean that a human brain organoid has some rudimentary form of consciousness (Sect. 4); I will also consider the studies conducted on human-animal chimeras carried out by implanting human brain organoids into the brains of mice (and in the future of other animals) (Sect. 5); I will address the topic of possible research on human brain organoids for the purpose of studying human consciousness (Sect. 6); and I will conclude with a brief general discussion of the topic of research with and on human brain organoids in the event that they may become conscious (Sect. 7).

## Human brain organoids: what are they?

One of the biggest and most promising advances in biomedical research over the last decade has been the development of organoid culture techniques. Organoids are three-dimensional cell cultures grown in a dish that recreate the spatial morphology, structural features, and physiological responses of the represented organ of origin, as well as some of its key cell types. To testify that we are dealing with a new frontier whose potential and implications are not yet well explored, there is the fact that the first published landmark study on intestinal organoids dates back to 2009 (Sato et al. 2009). The first study that has paved the way to cerebral organoids dates back only to 2013 (Lancaster et al. 2013; Lancaster and Knoblich 2014).

The differentiation between cerebral organoids and embryonic stem (ES) cells or induced pluripotent stem (iPS) cells can now generate three-dimensional in vitro cultures that mimic the developmental process and organization of the developing human brain (Setia and Moutri 2019; Arlotta and Paşca 2019). These so-called mini-brains have immediately provided a unique, physiologically relevant in vitro model system for the study of human neurological development and diseases. The advantage of this technique over 2D cell cultures is enormous. In general, organoids contain many different types of cells specific to the organ in which they are induced to develop, in order to reproduce the functionalities of the organ itself. This is achieved by using appropriate signaling factors that mimic the signaling environment typical of organ development in the human body. For this reason, organoids display a complex architecture similar to that observed in vivo: for example, HCOs have an appropriate cellular stratification.

Organoids have quickly found a wide variety of applications, from basic research to translational and industrial uses. First, organoids are providing important information on the development of the tissue that they model. Secondly, they also represent relevant models for studying cell biology, which includes tissue regeneration mechanisms and interactions with bacteria, viruses, and cells from other tissues. From an experimental point of view, organoids add up the advantages of high complexity of cell cultures with the absence of confounding variables typical of animal models and the ease of in vitro handling and lower costs in terms of resources and time. For this reason, in many cases (though not all), they can already complement or replace in vitro experiments that today use primary cells or immortalized cells and animal experimentations. The fact that organoids are genetically stable, i.e. they maintain the genotype and phenotype of the tissue of origin, allows them to be used as reliable models for the study of diseases and their mechanisms and progression. Organotypes are also being used to predict a patient’s specific response to a certain drug treatment. At least for some organs, it is possible to envision reaching, one day, the goal of what is considered the holy grail of biomedical research, namely the production of organs grown in a dish that can be transplanted into the body of a patient with full biological and immune compatibility, without needing a living donor.

A human cerebral organoid is grown in the lab starting from an embryoid (tissue that has some embryonic features) obtained from ES or iPS cells. The nervous system grows from the ectoderm layer of an embryoid. Ectodermal cells are placed into matrigel droplets (which provide nutrients) and floated in a nutrient broth in a rotating bioreactor. After 10 days the organoid develops neurons. After 30 days it displays regions similar to parts of foetus developing brains. Lacking vascularization and consequently blood supply, brian organoids can reach about 4–5 mm across and remain vital for a year or even more. For different scientific purposes, scientists grow 3D cell cultures systems of different complexity. They range from neurospheres (small clusters grown in suspension) to neural aggregates (based on pluripotent stem cells by first forming an embryoid body), up to cortical spheroids (containing only cortical neurons and astrocytes) and cerebral organoids or whole-brain organoids that are models derived from pluripotent stem cells capable of producing organized structures resembling those of the human brain.

By adding patterning factors, one obtains models of specific regions (e.g. the forebrain), while without patterning factors one obtains a complex structure representing multiple brain regions: a mini-brain, like the one first created by Lancastaer et al. (2013). This technique developed by Lancaster and colleagues and consistently perfected by other research groups (Velasco et al. 2019) has already been used for the study (and treatment) of many diseases, starting from microcephaly and Zika virus to Angelman’s disease and Hungtington’s disease. In just a few years, HCOs have been used to learn how viruses affect human neurodevelopment, to assess the effects of nerve tissue exposure to environmental toxins, to create genetic neurological disorders models and find possible remedies, and to have oncological models from which to understand the progression of cancer and find possible treatments (Li et al. 2016; Schwartz et al. 2015; Yin et al. 2016; Qian et al. 2016, 2017; Pacitti et al. 2019; Sun et al. 2019; Grenier et al. 2019).

These important clinical applications go hand in hand with the possibility of studying the brain in all its development stages in unprecedented ways. Brain organoids that have been grown for many months have reached important levels of differentiation and cellular activity. The small spheres that initially appeared to be only 3-dimensional transpositions of the cultures of nerve cells on the Petri dish have begun to show important functionalities. Just to name a few, Birey et al. (2017) produced “three-dimensional spheroids from human pluripotent stem cells that resemble either the dorsal or ventral forebrain and contain cortical glutamatergic or GABAergic neurons”, thus showcasing the saltatory migration of interneurons in the fetal forebrain. They also showed that after migration, interneurons functionally integrate with glutamatergic neurons to form a microphysiological system. And “spheroids cells were remarkably similar with those from corresponding regions of humans’ fetal brain”, with “both excitatory and inhibitory neuronal activity” (Camp and Treutlein 2017; cf. Pacitti et al. 2019).

It is said that without inputs and outputs, the HCOs’ neural networks cannot reach maturity, but the issue is open because “transcriptional analysis and comparison to the developing human brain have revealed that hCSs after 2.5 months resembled the mid-fetal prenatal brain (19–24 post-conception weeks). Cortical neurons were accompanied by a network of nonreactive astrocytes and were synaptically connected” (Paşca et al. 2015; Paşca 2018). Today, lab-made cerebral organoids already “acquire structural traits of mature neurons, including dendritic spine-like structures” and researchers have recorded excitatory spikes in organoids grown for 8 months, where monosynaptic connections were detected with high-density silicon microelectrodes (Quadrato et al. 2017). These findings “suggest that brain organoids establish neuronal networks that can support self-organized patterns of activity” (ibidem).

Also, HCOs show the differentiation of photoreceptor-like cells endowed with proteins for light responsiveness. These photosensitive cells “can respond to non-invasive, light based sensory stimulation” (ibidem). These steps forward indicate that it is possible to transmit afferent stimulations to cerebral organoids, and this has important implications, since so far one of the main limitations in the development of HCOs has been precisely the fact that they do not have any sensory communication with their environment. A further step forward has been made with new methods of cultivation of cerebral organoids (air–liquid interface) that have allowed to generate diverse nerve tracts with functional outputs (Giandomenico et al. 2019). In this way, “these cultures exhibit active neuronal networks, and subcortical projecting tracts can innervate mouse spinal cord explants and evoke contractions of adjacent muscle in a manner dependent on intact organoid-derived innervating tracts” (ibidem). In other words, cerebral organoids have proved capable of inducing movement, although not yet of a purpose-oriented kind.

A recent study showed for the first time that cortical organoids generated from induced pluripotent stem cells can spontaneously develop periodic and regular oscillatory network electrical activity which resembles the EEG patterns of preterm babies (Trujillo et al. 2019). This means that, even in the absence of external or subcortical inputs, 10-month-old HCOs can develop according to a specific genetic program, like all human beings, and manifest a complex brain activity. “The spontaneous network formation displayed periodic and regular oscillatory events that were dependent on glutamatergic and GABAergic signaling" (ibidem). The firing rate, up to two or three per second, and the kind of waves—gamma, alpha, and delta—are all a hallmark of a vital human brain. Indeed, a machine-learned model based on a preterm newborn’s EEG features was able to predict the organoid culture’s age based on the electrical activity of the organoid itself. In other words, the software found no significant differences in EEG between patterns of preterm babies and patterns of human cerebral organoids. This, however, does not mean that the two kinds of brains are the same.

In another study (Sakaguchi et al. 2019), researchers have managed to visualize activities in networks and connections between individual neurons in cortical spheroids. They managed to detect dynamic changes in the calcium ion activity and find comprehensive activities among cells capable of organising themselves into clusters and form networks with other nearby clusters. The manifestation of a synchronized neural activity can be the basis for various relevant brain functions, including memory. Another important element brought to light by research is that neurons grown in vitro fire spontaneously, which is one of the ways neurons grow and create new connections in the human brain.

## Theories of consciousness and potential consciousness of HCOs

Although the available techniques do not seem capable of producing cerebral organoids that mature beyond the equivalent of a prenatal brain (with tens of millions of nerve cells, while an adult brain has 86 billion of them), much research is focusing on overcoming the main limit of the current models, namely the absence of vascularization. It is known that blood vessels play a decisive role in gas exchange, nutrient supply, and waste removal. The lack of microglia, which in turn could be added from the outside, is also important. As we shall see, one way that has already been experimented to fix this problem is to transplant HCOs into mice to exploit the latter’s vascular system.

However, even in the current research situation, based on which the majority of scientists working with organoids are convinced that they have not and cannot develop forms of consciousness, ethical questions in this regard have already been raised (Cheshire 2014; Bredenoord et al. 2017; Munsie et al. 2017; Lavazza and Massimini 2018a; Farahany et al. 2018; Lavazza and Massimini 2018b; Boers et al. 2018; Lavazza 2019; Hostiuc et al. 2019; Sawai et al. 2019; Lavazza and Pizzetti 2020). A more pressing, albeit non-detailed, call for caution was raised in October 2019 in Chicago at the annual meeting of the Society for Neuroscience. Elan Ohayon, the director of the Green Neuroscience Laboratory in San Diego, California, and his Toronto-based colleagues Ann Lam and Paul Tsang stated that “if there’s even a possibility of the organoid being sentient, we could be crossing that line. We don’t want people doing research where there is potential for something to suffer” (Sample 2019).^1^

On that same occasion, Ohayon and colleagues presented a paper called “A computational window into the problem with organoids: Approaching minimal substrates for consciousness”. Their work was about “several computational network models and accompanying methods for analyzing dynamics that may help identify the encroachment toward functional and possibly sentient activity” in reference to human brain organoids. And, according to the authors, “assessment informed by the models and associated dynamics suggests that current organoid research is perilously close to crossing this ethical Rubicon and may have already done so”. Nevertheless, they underlined that “it is important to note that the observations in this computational study point at minimal guidelines and undoubtedly would fail to identify alternate forms of sentience”. Hence the demand to discontinue research with human brain organoids implants in non-human animals and in all those cases where there is a reasonable chance that HCOs might become sentient.^2^

The key point is the identification of the underlying mechanisms and minimal conditions for consciousness. According to Ohayon and colleagues, “there are at least five domains that can help anchor this question when studying the brain: [1] compositional (e.g., atomic, molecular), [2] causal (e.g., genetic, evolutionary), [3] anatomical (e.g., cellular, network geometry, brain regions), [4] physiological (e.g., cellular, network, whole brain activity), and [5] behavioral (e.g., embodied, virtual)”. So far, however, all attempts to find necessary and sufficient specific correlates of consciousness seem to have failed. Therefore, in relation to HCOs it may be useful to consider at least two of the most promising scientific theories of consciousness. It is not possible here to give an in-depth and complete description of them, nor to consider the objections that have been made against these theories. I will therefore proceed to a general overview of them.

The two best-known theories are also those competing in the first round of the Templeton Foundation contest mentioned above: the Theory of Global Workspace (GWT), supported by Stanislas Dehaene, and the Integrated Information Theory (IIT), proposed by Giulio Tononi. In a nutshell, GWT states that the prefrontal cortex of the brain, which controls higher order cognitive processes such as decision making, acts as a central processor that collects and prioritizes information coming from the senses. It then transmits this information to other areas of the brain that perform additional tasks. Dehaene thinks that this process of brain selection and sharing is what we call consciousness.

On the contrary, IIT assumes that consciousness derives from the interconnection of brain networks. The more neurons interact with each other in a complex and non-stereotypical way, the higher the level of consciousness, even without any sensory input. IIT advocates hypothesize that this process takes place in the back of the brain, where neurons connect in a grid structure. For this reason, a sort of *experimentum crucis* will involve researchers measuring the neuronal response of a person when they become aware of an image. GWT predicts that the anterior part of the brain will suddenly become active, while IIT posits that the posterior part will be constantly active. This quick outline concerns research on healthy adult humans and allows us to understand the positions at stake. Considering in more detail the two theories will allow assessing their potential relevance with respect to human cerebral organoids.

### The integrated information theory

The Integrated Information Theory (IIT) claims that if a system has the intrinsic potential to integrate information, then we can assume presence of consciousness (Tononi et al. 2016). Indeed, according to IIT, an experience does not require the involvement of body and world, language, introspection or reflection: it can exist without spatial frames of reference or a sense of the body and the self, and it is not reduced to attention or memory (Koch et al. 2016). More precisely, Tononi and colleagues argue that information coming from the environment is an important element for the brain, shaping the quality and structure of the conscious experience we have through the evolutionary history of the human brain (Joshi et al. 2013) as well as through the individual’s development and plastic changes (Tononi and Sporns 2003; Tononi and Koch 2008). However, the brain as such, or even better a precise conceptual structure within it, i.e. cause-effect structure, can be the only sufficient condition for consciousness. In this respect, Tononi and colleagues make the example of a dreaming brain as isolated from the external world, in a body completely paralyzed and unresponsive, but still producing a world of conscious experiences (Nir and Tononi 2010; Sarasso et al. 2014; Tononi 2012).

Thus, consciousness as integrated information is intrinsic and solipsistic, and it does not necessarily require any extrinsic relation outside the system, nor functions or purposes towards something else (Tononi 2008). Indeed, a system with the right architecture for a sufficient integration of information could exist in complete detachment from the external world, producing its own internal states (Oizumi et al. 2014). Furthermore, according to IIT, the brain is a sufficient non-necessary condition for consciousness, since, in principle, any artificial or natural being which integrates information can be conscious, even without a brain (Oizumi et al. 2014; Tononi and Koch 2015).

### Global workspace theory

The Global Neuronal Workspace Theory (GWT) is a cognitive theory of consciousness with neuronal correlates, according to which the latter is tantamount to global information availability (Dehaene and Naccache 2001). The theory argues for the existence of recurrent and competitive top-down/bottom-up loops that accumulate information from perceptual, motor, attention, memory, and value networks, then sharing and broadcasting back information to lower-level processors. This broadly distributed network permits conscious access to the unified and synthesized information, i.e. consciousness of some specific content (Dehaene et al. 2011).

This theory does not claim for the importance of some specific brain area, because it is the global state of information availability as such, i.e. neuronal workspace through long-range cortico-cortical fibers, that is necessary for consciousness. According to GWT, a neuronal marker useful to determine the level of information availability is the late component P300, particularly P3b (in fact, P3a component may reflect automatic and non-conscious attention) (Dehaene et al. 2011). P3b is observed mainly during subjective reports and the diffused and simultaneous involvement of multiple area activations. According to the theory, the world plays an “accessory role”, since consciousness can be mainly described as a process of unification, selection and broadcasting of information, i.e. “brain-wide information sharing” (Dehaene 2014). The world can be understood as the set of stimuli that comes into the brain through the sensory system and is processed by a multitude of unconscious processes, in order to produce a faithful representation of the environment for the following high-cortical processing, i.e. conscious access (Dehaene et al. 2006).

Whereas in some theories of consciousness, like the Temporo-spatial Theory (Northoff and Huang 2017), the world is constitutive as a necessary predisposition for consciousness, in the two theories I have described (IIT and GWT), the world plays a somehow secondary role, being useful for the development of the brain and for the enrichment of the subjective experience, but consciousness could arise even without it, purely through brain activity. And this element leads to an important distinction between the accessory and constitutive role of the world for consciousness, which is relevant if one wants to assess the possibility that a cerebral organoid develops some form of consciousness without structured relations with the external environment.

## Presence of consciousness and moral status

There are two questions about the presence of consciousness in HCOs and their possible moral status. The first is strictly philosophical and concerns how to identify what consciousness is and what characteristics attribute moral status to an entity, while the second is gnoseological and concerns how consciousness can be found and evaluated and the characteristics that allow moral status to be given. In the case of cerebral organoids, however, moral status is linked to the presence of forms of consciousness, so that the identification of the characteristics capable of motivating the attribution of moral status derives from the presence of evidence of consciousness.

The descriptions above show that the current structural impossibility of HCOs to relate to the external environment excludes that they can be conscious in the sense envisioned for example by the Temporo-spatial Theory. This means that in the absence of verbal reports or behavioral manifestations, the detection of consciousness must rely on parameters that can be acquired with instruments such as an EEG, the interpretation of which also depends on the theory of consciousness that we believe to be the most scientifically founded.

Based on IIT, instead, Lavazza and Massimini (2018a, b) have hypothesized that it can be possible to measure consciousness also in human cerebral organoids. The Integrated Information Theory posits two phenomenic axioms that give rise to postulates on the properties of brain mechanisms that support consciousness. The axioms are: (i) conscious experience is informative (each conscious experience differs in specific ways from countless other possible experiences); (ii) conscious experience is integrated (no conscious experience can be divided into parts). It follows that a system has subjective experience to the extent that it is able to integrate information. This capacity depends on an optimal balance between differentiation (information) and unity (integration)—a non-trivial condition for a physical system. Indeed, at first sight, that these two properties appear extremely difficult to reconcile.

In this sense, IIT proposes a theoretical measure (PHI) and empirical metrics to quantify the ability of a system to integrate information. The Perturbational Complexity Index (PCI) is a parameter inspired by the main postulate of IIT, namely that consciousness is based on the joint presence of integration and differentiation in the brain. The calculation of the PCI locally involves perturbing the cerebral cortex (by transcranial magnetic stimulation, TMS) and measuring the complexity of the electrical response in the rest of the brain (by EEG) (Massimini et al. 2009; Casali et al. 2013; D’Andola et al. 2018).

The basic idea is that the PCI is low if the interactions between neuronal elements are reduced (loss of integration), because the response induced by TMS is limited in space. The PCI is low even if many connected areas react to the perturbation, but they do so in a stereotyped way (with a loss of differentiation), because in this case the response is wide but not complex. The PCI should only reach high values if the initial disturbance is transmitted to a large network of neuronal elements that react in a differentiated way. As such, the PCI is independent of sensory processing, executive function, or motor behavior. For this reason, with specific technologies, it could also be applied to cerebral organoids.

However, although this technique has been tested on comatose and vegetative patients with instrumental results consistent with behavioral evidence (Casarotto et al. 2016; Gosseries et al. 2014; Owen 2017), there is no consensus that IIT captures the real mechanism of consciousness (Doerig et al. 2019). For this reason, it makes sense to turn to the Global Workspace Theory, which like IIT does not exclude that there can be consciousness even in the absence of direct and constant interaction with the external environment. Nevertheless, its application to the case of HCOs seems to immediately highlight the problem of their reduced size. In fact, for GWT, long-range cortico-cortical functional connectivity is a necessary condition of conscious state (Bourdillon et al. 2019). In HCOs with a size of a few millimeters these (centimeter) long connections are absent and, therefore, the presence of forms of consciousness would seem extremely unlikely.

Assuming, however, that there is a reasonable likelihood that in the near future a human cerebral organoid will be capable of a form of sentience, what ethical implications would arise from this? For example, even if it is true that the brain has no pain sensors and therefore neither do HCOs, it may still be possible for an isolated brain to experience something like pain without having any body parts. Phantom pain following arm or leg amputations is an example of how pain can be experienced by the brain, even though it is no longer connected to the body part or sensitive nerve fibres where the pain impulses would normally occur. Under this assumption, it would be possible to measure electrical activity in those areas of the brain that are usually particularly active when experiencing a painful sensation (although the phenomenon of phantom limb pain may be due to having actually had an arm or leg).

Now, based on a shared concept of it, moral status is a condition for which a certain entity receives consideration in the ethical sphere for being that entity and not only for its relations with other entities (Jaworska and Tannenbaum 2018). Moral status is not equivalent to moral value, the former being a basic condition that does not determine the degree of the latter. Moral status, in fact, is attributed on the basis of a being’s intrinsic properties and, for living beings, can specifically be attributed to entities that have subjective interests, i.e. interests in having or not having specific subjective experiences and in not being harmed in a general sense.

In this vein, subjective interests are generally linked to certain subjective experiences, or in some cases to the potential capacity to have subjective experiences, as it happens for unconscious born persons to whom are recognized legal rights and protection. However, we need to distinguish among *subjective interests*, which are primarily in the moral domain and depend on the agreement of a specific community on some criteria, and *subjective rights*, which are established as duties for the agents who interact with the entity vested in those rights. So, to obtain moral status it is necessary to have (or to have had or potentially have) some form of consciousness. Some may want to endow with a moral status a corpse as well. We obviously respect the dead, but their moral status seems not to be unconditioned and have different criteria.

Secondly, moral status can be attributed based on various justifications, the most relevant of which include having a certain relevant moral characteristic or having a relationship of similarity or biological affiliation. In the case of HCOs, the necessary premise seems to be the possession of subjective interests and, therefore, of some even minimal form of consciousness, i.e. sentience. If cerebral organoids possessed this characteristic, this would not qualify them as moral as such: they would probably have to reach a certain threshold of complexity, as happens in the heated and controversial debate on which states of consciousness a human being must manifest in order to be defined as a *person*, who is in general deemed as the maximum degree of moral status. On the other hand, HCOs are human by definition, as they come from human cells, and this biological affiliation could grant them a moral status. Now, the biological criterion is contested as speculative and discriminatory in relation to other species. But the argument against the criterion of biological belonging is that other species also have sentience and therefore subjective interests. It seems, therefore, that having sentience could guarantee cerebral organoids a moral status.

What is important to underline is that once an entity has been given moral status, this does not imply any necessary consequences. First of all, one may consider that there are different levels of moral status, which can be for example partial or complete, or that moral status is a continuum, based on the characteristics and complexity of the psychic life of the entity under consideration (Streiffer 2007). In fact, one must specify the moral hierarchy in which the entity is inserted, what kind of rights it acquires through its new status and what kind of obligations, if any, other moral agents have towards it. This is based on the premise that obtaining a moral status in itself neither implies the acquisition of specific rights nor imposes specific obligations on other moral agents. The moral status attributed to a sentient entity can then be further broken down analytically into two components. The first concerns a purely evaluative function that gives an intrinsic value to the entity granted moral status. The second concerns a prescriptive function, according to which the entity with intrinsic value has the right to receive specific treatment by moral agents.

It would therefore seem plausible to attribute a moral status to any human whole-brain organoids (or perhaps even to cortical spheroids) that might experience sensations capable of generating subjective interests, such as pain or suffering of some kind (caused by the experimental situation, for example by the induction of malformations, diseases and pathological development for study purposes). And if larger HCOs manifest higher forms of awareness, their moral status might be accompanied by higher moral value compared to organoids of lower cortical complexity.

The cases of human embryos and foetuses—which certainly do have a moral status (as human and/or as potentially sentient entities), but which under some legislations up to 14 days are legally used for experiments or whose gestation may be interrupted at different times according to different national laws—indicate that the attribution of moral status is not tantamount to the unavailability of such entities to research (cf. Chan 2018; Pera et al. 2015). In those cases, while moral disagreement remains strong, many if not the majority of people believe that embryos or foetuses have a lower moral value than fully conscious individuals, and that other moral values prevail in relation to their use, such as scientific research in favour of seriously ill people or the decision-making autonomy of mothers. However, there are reasons to doubt that one can establish a full analogy between foetuses and HCOs, as the latter would only have their own moral characteristics if they developed some form of consciousness. In fact, they are neither physically autonomous nor able to give rise to an adult human being. Yet the brain is the key organ of the person, the one from which one can deduce the presence of life in a person and which, if conscious, even though in a dish, should be considered a person, with increasing moral value the greater its consciousness.

## Cerebral organoids and chimeras: risks and limitations

A recent interesting proposal on the moral relevance of being human may help to understand why the analogy between HCOs that have developed even rudimentary forms of consciousness and other cases already discussed in bioethics (embryos or fetuses) is not adequate. And it may also help in considering the issue of the implantation of human cerebral organoids in non-human animals, both in order to make HCOs develop more than what is currently possible in a dish, and to enhance the receiving non-human animals.

In this regard, Kipke (2019) proposes to overcome the classic dichotomy between personism and speciesism in the debate on the moral status of human beings and the boundaries of the moral community. As we have already seen, personism considers certain mental properties morally crucial, whereas speciesism considers membership in the human species morally crucial. The idea is to find a new criterion that is in line with many common moral insights, which are at odds with the traditional approaches of personism and speciesism (for example, the exclusion from the scope of maximum moral protection of people with altered states of consciousness or of some animals interacting significantly with human beings).

We recognize each other quite directly, through the *living human gestalt* or *the form* of the body. We recognize ourselves as living physical beings with a specifically formed human body. Despite all the differences, whether baby or old man, small or tall, thin or fat, beautiful or ugly, disabled or non-disabled, of whatever ethnic background or gender—we immediately recognize ourselves as beings with an undoubtedly human body (Kipke 2019).

In this sense, “the human form is what makes us equals” and is a “morally significant property that all humans possess equally”. As the essential criterion to belong to the moral community, having a living human form has significant moral consequences. “Newborns, as well as demented people and the severely mentally disable, belong to us as a matter of course” (Kipke 2019). However, this inclusive morphological approach does not include, for example, early-stage embryos, which do not exhibit the human form as they are faceless and invisible to naked eye. Now, it is not necessary to fully subscribe to this new approach, which seems to have some inconsistencies within it, to understand that the case of human cerebral organoids does not fit with any of the assumptions of this theory. In fact, HCOs do not have a human form and are not visible to the naked eye but could have those qualities that, according to Kikpe, are “evaluatively and normatively significant in persons”, namely a formed consciousness capable of “understanding themselves as members of a moral community”.

Human cerebral organoids thus seem to elude any typical characterisation and this is further accentuated in the moral debate on chimeras, which are “single organism[s] made up of cells of different embryonic origins” (Nagy and Rossant 2002). The first study involving human tissue dates back to 1969, with a tumor grafted into a mouse (Rygaard and Poulsen 1969). Here, I’m only interested in non-human animals with human neural tissue, since scientists are trying to produce chimeras to make HCOs vascularize and grow better than in vitro. But there is also the possibility of enhancing animals with human nerve cells.

A vascularized and functional in vivo model of brain organoids was recently obtained thanks to a method for transplanting human cerebral organoids into the adult mouse brain (Mansour et al. 2018). “Organoid grafts showed progressive neuronal differentiation and maturation, gliogenesis, integration of microglia, and growth of axons to multiple regions of the host brain. In vivo two-photon imaging demonstrated functional neuronal networks and blood vessels in the grafts. Finally, in vivo extracellular recording combined with optogenetics revealed intragraft neuronal activity and suggested graft-to-host functional synaptic connectivity” (ibidem). In an earlier study, researchers “engrafted human glial progenitor cells into neonatal immunodeficient mice. Upon maturation, the recipient brains exhibited large numbers and high proportions of both human glial progenitors and astrocytes” (Han et al. 2013). The outcome was that “long-term potentiation was sharply enhanced in the human glial chimeric mice, as was their learning, as assessed by Barnes maze navigation, object-location memory, and both contextual and tone fear conditioning. Mice allografted with murine glial progenitor cells showed no enhancement of either long-term potentiation or learning” (ibidem).

HCOs developed within a non-human animal could hybridize cognitively, because they would rely, for example, on the sensory apparatus of the host animal, which is different from the human one and therefore gives a different perspective on the world from that of a brain grown together with a human body. This would mean that we would have an entity with no human form that does not belong completely to the human species, thus excluding that it can be attributed a full moral status. However, from the point of view of personism, i.e. the presence of mental states, we would be dealing with hybrid states that are difficult to evaluate, even if their very presence seems to imply a "mixed" moral status between those of human beings and those of non-human animals.

Assuming that an HCO grown in the organism of a non-human animal and integrated at least in the vascular system of the host could no longer be transplanted in vital form, it would seem to be ethically highly problematic to conduct such experiments when there is a reasonable chance that human cerebral organoids of this type might develop some form of sentience or consciousness. A conscious organoid forced to exist inside a non-human animal could be deprived of part of its dignity, as it would be in the hypothesis of a perfectly formed human being of a below-average size who was coupled, without the possibility of release, to a mouse or a monkey (think of the case of the woman connected to a violinist proposed by Thomson (1971) to argue against the obligation to remain connected if the union was made against the will of the subject).

In the case of non-human animals that develop new cognitive abilities through human nerve cell implants (e.g. cortical spheroids), i.e. increase the range of their mental states, somehow "humanizing" their consciousness, these types of experiments should be considered highly problematic if they aim, for example, to make the non-human animal a better source of organs for transplantation or use it as a guinea pig for experiments that cause suffering or death. If, on the other hand, the goal was to see the effect of cognitively "humanizing" certain species genetically close to the human being, one might wonder whether experiments of this kind would not be detrimental to the dignity of these new animal entities, unless they were treated as fully-fledged human beings, for example as severely disabled persons. It would seem plausible to defend the idea that anything human should be treated in such a way that it is never a means and always an end, even if the mental abilities it exhibits are not those typical of a healthy adult (see Sect. 6).

Several proposals have been made to address ethical concerns about chimeric research. Koplin and Savulescu (2019a) aim at regulating part-human chimera research in order to balance ethical research with the prevention of unethical experiments involving the use of chimeras with an uncertain—or potentially substantial—degree of moral status. In the range of possible regulatory frameworks, Koplin and Savulescu (2019a) suggest adopting a moderately restrictive approach, which “allow[s] the full development of chimeras with humanized brains, but prohibit[s] experimentation until after the chimeric animal’s moral status has been determined”.

Mann et al. (2019), instead, propose a commensurability approach, “which states that regardless of how a chimera came into existence or what species it belongs to, the chimera should not be treated in a way that is incommensurate with its moral status”. According to its proponents, this principle depends, in its practical application, on the attribution of full or partial moral status and on the rights and duties deriving from it. However, it is interesting to note that their conclusion coincides with the position suggested here with regard to experiments aimed at studying the emergence of human consciousness.

Because of the need to treat creatures commensurate with their moral status, creating chimeric models with advanced human-type brain functions that ground full moral status would in some ways be self-defeating. The same reasons that prohibit certain kinds of research on humans would also apply to them. Chimeras with enhanced cognition immediately below this level will require stringent justification for use as research subjects (Mann et al. 2019).

In this sense, the use of chimeras for the sole purpose of acquiring new knowledge about the mechanisms of consciousness seems to clash with strong, and potentially insuperable, ethical objections related to the instrumental use of entities which, precisely because of their moral status, acquired on the basis of sentience and cognitive abilities, can only be considered ends and not means in a moral perspective.

## Studying the consciousness of human cerebral organoids?

Hence the double-edged sword character of consciousness research through human cerebral organoids. On the one hand, the latter could become an extraordinary tool to penetrate the secrets of consciousness, but on the other hand, the very appearance of consciousness in them should constitute an impassable ethical limit to the use of HCOs as means for this purpose.

Why can’t persons be treated as means? The classical Kantian justification refers mainly to the concept of autonomy. The point, for the purposes of our discussion, is that first we need to be able to talk about human cerebral organoids as persons, and only then can we introduce arguments that prohibit the instrumental use of an entity that is granted the full moral status of personhood. In the contemporary debate, for example, according to DeGrazia (2007), personhood “is associated with a cluster of more specific properties without being precisely analyzable in terms of any specific subset: autonomy, rationality, self-awareness, linguistic competence, sociability, moral agency, and the capacity for intentional action”. But these are not necessary and sufficient conditions: in fact, “a person is someone who has enough of these properties”.

In this perspective, the emergence of sentience or a minimum degree of consciousness would not give HCOs the status of a person nor, consequently, a full moral status by virtue of which to have rights such as to guarantee their absolute protection, as is typically the case for adult human beings. However, the idea of studying the mechanisms of consciousness of an adult human being, therefore of a "person" from the moral point of view, with cerebral organoids developed to that degree seems to place the entire process of creating conscious HCOs, whatever their current state of sentience or awareness, within the context of treating an entity with moral status as a pure means. This eventuality seems contrary to a strong normative rule that can be easily rationally justified—indeed, it might even be considered intuitively self-evident.

In justifying treating persons as ends and not as a means in a non-strictly Kantian way, Audi (2016, pp. 88–90) resorts to the concept of pain. We can in fact think of not harming as the least form of respect and recognition of dignity that is due to an entity endowed with moral status. Not harming can be understood as not causing negative sensations (i.e. those sensations that we generally try to avoid, albeit to different degrees and with exceptions). Accordingly, pain can be the guideline to implement the prescription not to use as means those entities to which we recognize some degree of moral status related to their ability to have subjective experiences.

Since pain is a subjective sensation, in the case of non-human animals we make inferences from manifest behaviour. In some cases of phylogenetically ancient species we may think that certain behaviours are only avoidance reflexes and do not have an internal equivalent similar to the phenomenology of pain typical of human beings. In this sense, the "pain" relevant here is understood as something that has a certain effect and therefore can be associated with a minimal form of consciousness. HCOs, being similar to adult human brains, are likely to develop the ability to experience this form of pain.

The topic of pain is part of the discussion of how to treat human persons, but the attitude that we should have in the face of the suffering of a sentient entity can be the basis for a more general mode of moral behavior, although we don’t consider that entity as a person. According to Audi, the notion of pain is psychological: pain is a psychological phenomenon that has behavioral aspects (avoidance, for example) as well as introspectable ones (the feeling that is experienced). In general, we can understand the notion and identify the phenomenon without having nor applying a moral concept of it. However, suffering appears to require a palliative response, one that either attenuates or interrupts it. This makes this response fitting and as such it involves behaviour that treats the entity as an end. This type of response is fully justified and signals the fittingness of treating the entity as an end. In this sense, giving an a priori justification for palliative acts, such as cooling a burnt hand, pain is moral and therefore normative in its consequences.

This ability of pain to provide reasons to act gives it a strong normative significance, such that it makes it appropriate to speak of the moral being of something in terms of the result or consequence as a type of normativity. And this is particularly appropriate when the element that provides the reasons to act can be considered self-evident, as in the case of pain. In Audi’s view, the concept of pain is neither axiological nor normative in any respect. Its normativity is not internal to the content of the concept but is a consequence of its application. The concept of pain is psychological and descriptive, but since the fact that some entity is in pain provides—and also implies a priori—a reason to do something to eliminate its suffering, it has a significant relationship with normativity "proper", i.e. with what is constitutively normative. In fact, Audi states: “If no one could suffer pain, morality would be quite different from what it is”.

In this sense, it does not seem ethically permissible to use human cerebral organoids as pure means if they are sentient or endowed with some rudimentary form of consciousness, i.e. able to experience suffering, even if they are not persons in the full sense of the word, precisely on the basis of the aforementioned argument related to the normativity of pain that should be alleviated.^3^ If it is true that current biotechnologies seem to “blur the legal distinction between human beings and other living organisms, between living human beings and dead ones, and between human tissues and cells and nonhuman ones” (Knoppers and Greely 2019), then sentient HCOs would “substantially” (“which is not measurable by percentages or similar specific tests but will be a judgment call”) fall within the category of “human”, with the resulting corollary of rights.

On the other hand, we know that human beings who may not have full moral status under restrictive criteria of personhood, like individuals in a vegetative state, are still not used as means. It is not considered morally acceptable to use them as guinea pigs for experiments, although for some philosophers and bioethicists it is legitimate to end their existence.^4^ In the case of sentient human cerebral organoids, we should also consider the fact that they are voluntarily and specifically produced by us for the purpose of being instruments of experimentation, which aggravates their condition of exploitation.

It can be argued that non-human sentient animals, perhaps endowed with rudimentary forms of consciousness, are still used in the experimental field, as they are not granted a full moral status, although today this is generally done only when strictly necessary, while trying to minimise their suffering. The fact remains, however, that pragmatic speciesism is implicit in these practices. Non-human animals are used as means for research aimed at the welfare of human beings—and this research is as such justified—but the reverse never occurs, and human beings are never used as means for the purpose of animal welfare. If there is a justification for these practices, it amounts to the fact that the human species is considered to have a higher moral status than the non-human animal, which can thus serve as a means. Alternatively, from a utilitarian point of view, it is considered that promoting the welfare of human beings entails a higher overall utility than that of non-human species.

In this perspective, human cerebral organoids that are sentient or endowed with some form of consciousness (and also chimeras that have developed an increased consciousness thanks to human grafts) are to be treated as ends and not as means, particularly for the study of the emergence and mechanisms of consciousness. Their moral status could even be considered superior to that of an embryo up to 14 days old, which many legislations do not protect against instrumental use or destruction for research purposes. The fact remains, however, that an embryo can develop into an adult human being, something that an HCO is precluded from doing, and this might result in the absolute protection of human embryos as well (Marquis 1989).

## Conclusion

We have seen how the technology that has led to the growth of human cerebral organoids in the laboratory is rapidly advancing (Benito-Kwieciński and Lancaster 2019) and how HCOs are one of the most promising revolutions in biomedical research. Despite the aforementioned limitations the most recent overviews highlight the advanced state of the art. “Human pluripotent stem cell-derived brain organoids produce a diversity of cell types that interact with each other in a complex 3D environment. Combining organoids resembling distinct areas into assembloids can be used to model aspects of interactions that occur between regions in the human brain. Organoids can be supplemented with non-central nervous system-derived cell types, including microglia and endothelial cells, to study the interplay of nervous system cells with immune cells and blood vessels” (Marton and Paşca 2019).

In addition, Xiang et al. (2019) “reported establishment of reciprocal thalamocortical projections. As the thalamus is the gate of all sensory input to the cerebrum, such a finding might have the potential to transmit sensory information to the neuronal tissue of the organoid. If the fused organoids are further fused with dorsal spinal cord and peripheral nerves, the organoid might have somatic sensory experience. If such fused organoids are further fused with neural retina and optic nerve tissues, they might recognize light” (Sawai et al. 2019).

These developments open up the possibility that HCOs may manifest a form of sentience or a more advanced degree of consciousness with the specific qualities typical of the human being in the growth phase, if not of a healthy adult once organoids are able to develop beyond the current limits. This characteristic of cerebral organoids makes them morally special, even though they cannot be considered persons in the full sense. Even considering HCOs as entities with a unique ontological status that needs to be clarified, they certainly share two convergent criteria for the attribution of moral status: the fact of potentially having a rudimentary form of consciousness and the fact of being part of the human species. If we combine this with the form of prudence that leads to “err on the side of generosity when resolving uncertainty regarding brain organoids cognitive capacity and/or moral status” (Koplin and Savulescu 2019b), it can be said that, once it is reasonably assumed that they meet the sentience threshold which makes them worthy of moral status, HCOs should enjoy a form of protection that is particularly restrictive with respect to their use as pure means.

Specifically, the use of cerebral organoids—if developed to very advanced stages precisely to study the emergence of human consciousness and its mechanisms—would end up violating Kant’s humanity formula in the extended formulation proposed here, which requires not using certain entities as means but only as ends. In this sense, even the mere culture of HCOs in the laboratory to do research on human consciousness would amount to a similar violation. In fact, even the recognition of a minimal form of moral status combined with Kant’s proviso induces to spare such an entity forms of suffering that it could experience as a sentient being. Does this mean that human cerebral organoids should no longer be used for biomedical research? Certainly not. The discriminating point is the potential (as we cannot be sure so far) presence of more complete forms of sentience or consciousness, whose verification is undoubtedly complex and controversial, but whose possibility can no longer be ignored at the present state of the art.

Koplin and Savulescu (2019b) have proposed to make the use of HCOs proportionate to some critically important purposes or sufficiently great expected benefits of the research. This view implies the lawfulness of using both “conscious or potentially conscious brain organoids (equivalent to 20 weeks’ in vivo brain development or more)” and “brain organoids with the potential to develop advanced cognitive capacities (e.g., mature brain organoids capable of interacting with the outside environment”. This framework to regulate the use of HCOs capable of developing higher consciousness and cognitive abilities is based on a consequentialist perspective that seems to admit a limited exploitation of human cerebral organoids in exchange for great expected benefits related to biomedical research.

What I have considered in this paper concerns specifically the research that could be undertaken for the study of the emergence and mechanisms of human consciousness, i.e. one of the most important scientific questions that still lacks a well-founded and shared answer. This objective appears to be achievable only with the growth of cerebral organoids able to progressively reach advanced stages of consciousness. This purely instrumental use of entities that would then acquire a high, or perhaps fully human, moral status seems unethical. Although we don’t know exactly what it would be like to be "a brain in a vat", as in the well-known mental experiment proposed by Putnam (1981), it seems reasonable to think that a conscious human cerebral organoid in a vat would be somehow similar to a person suffering from locked-in syndrome. And although there have been cases of people who have been able to claim not to be unhappy in such a condition (Owen 2017), it would not be morally licit to grow on purpose a series of human entities that experience that condition just in order to better understand how this happens.

## Footnotes

1 That claim is surely vague and seems to ignore that non-human animals suffer when they are experimented on. But I quoted Ohayon’s statement as it indicates the ethical relevance organoid research has acquired.

2 It is worth mentioning that other scientists at the same conference offered different data. Arnold Kriegstein presented single-cell RNA-sequencing results from both HCOs and human brain tissue. “Images of brain tissue contrasted with organoids clearly show the reduced complexity of the model brains, with fewer cell types and a different developmental timeline. Kriegstein showed that the organoid cells are under a type of cellular stress that seems to limit their ability to mimic normal cells” (MacKenzie 2019). And Paola Arlotta noted: “This is not an adult brain that you make. It’s not even a complete younger brain, it’s very primitive and reductive. There is a limit to what you can do in culture; they only grow to a certain size and they only make certain cells” (ibidem).

3 One can ask if I am here considering the “capability” or the “potentiality” to feel something (or to experience suffering) in order to provide grounds for the argument. My point is that in the case of HCOs the difference is small. If an entity has the capability to suffer and we do not want it to suffer, we should not do anything that can hurt it. The “potentiality” is related to our ignorance of the real inner functioning of HCOs. But if we reasonably suspect that an HCO can have some form of consciousness, then we can draw a legitimate inference that it can suffer as most living beings.

4 Obviously, most if not all legislations recognize legal status—and therefore a moral status—also to humans who are not capable of experiencing pain, including individuals in a vegetative state. This is precisely because they were conscious persons (and could become conscious again). It is known however that a number of biomedical scholars and bioethicists disagree on that and claim that only an (even though not perfectly) autonomous and rational individual can be considered as a person.

## References

[CR1] Arlotta P, Paşca SP (2019). Cell diversity in the human cerebral cortex: From the embryo to brain organoids. Current Opinion in Neurobiology.

[CR2] Audi R (2016). Means, Ends, and Persons. The Meaning and Psychological Dimensions of Kant’s Humanity Formula.

[CR3] Bayne T, Seth AK, Massimini M (2019). Are there islands of awareness?. Trends in Neurosciences.

[CR4] Benito-Kwieciński S, Lancaster MA (2019). Brain organoids: Human neurodevelopment in a dish. Cold Spring Harbor Perspectives in Biology.

[CR5] Birey F, Andersen J, Makinson CD (2017). Assembly of functionally integrated human forebrain spheroids. Nature.

[CR6] Boers SN, van Delden JJM, Bredenoord AL (2018). Organoids as hybrids: Ethical implications for the exchange of human tissues. Journal of Medical Ethics.

[CR7] Bourdillon P, Hermann B, Guénot M (2019). Slow cortico-cortical connectivity (2–5Hz) is a new robust signature of conscious states. BioRxiv.

[CR8] Bredenoord AL, Clevers H, Knoblich JA (2017). Human tissues in a dish: The research and ethical implications of organoid technology. Science.

[CR9] Camp GJ, Treutlein B (2017). Advances in mini-brain technology. Nature.

[CR10] Casali AG, Gosseries O, Rosanova M (2013). A theoretically based index of consciousness independent of sensory processing and behavior. Science Translational Medicine.

[CR11] Casarotto S, Comanducci A, Rosanova M (2016). Stratification of unresponsive patients by an independently validated index of brain complexity. Annals of Neurology.

[CR12] Chan S (2018). How and why to replace the 14-day rule. Current Stem Cell Reports.

[CR13] Cheshire WP (2014). Miniature human brains: An ethical analysis. Ethics & Medicine.

[CR14] D'Andola M, Rebollo B, Casali AG (2018). Bistability, causality, and complexity in cortical networks: An in vitro perturbational study. Cerebral Cortex.

[CR15] DeGrazia D (2007). Human-animal chimeras: Human dignity, moral status, and species prejudice. Metaphilosophy.

[CR16] Dehaene S (2014). Consciousness and the Brain: Deciphering How the Brain Codes Our Thoughts.

[CR17] Dehaene S, Changeux JP, Naccache L, Dehaene S, Christen Y (2011). The global neuronal workspace model of conscious access: From neuronal architectures to clinical applications. Characterizing Consciousness: From Cognition to the Clinic?.

[CR19] Dehaene S, Naccache L (2001). Towards a cognitive neuroscience of consciousness: Basic evidence and a workspace framework. Cognition.

[CR18] Dehaene S, Changeux JP, Naccache L (2006). Conscious, preconscious, and subliminal processing: A testable taxonomy. Trends in Cognitive Sciences.

[CR20] Doerig A, Schurger A, Hess K (2019). The unfolding argument: Why IIT and other causal structure theories cannot explain consciousness. Consciousness and Cognition.

[CR21] Farahany NA, Greely HT, Hyman S (2018). The ethics of experimenting with human brain tissue. Nature.

[CR22] Gennaro RJ (2018). The Routledge Handbook of Consciousness.

[CR23] Giandomenico SL, Mierau SB, Gibbons GM (2019). Cerebral organoids at the air-liquid interface generate diverse nerve tracts with functional output. Nature Neuroscience.

[CR24] Godfrey-Smith P (2016). Other Minds: The Octopus, the Sea, and the Deep Origins of Consciousness.

[CR25] Gosseries O, Di H, Laureys S (2014). Measuring consciousness in severely damaged brains. Annual Review of Neuroscience.

[CR26] Grenier K, Kao J, Diamandis P (2019). Three-dimensional modeling of human neurodegeneration: Brain organoids coming of age. Molecular Psychiatry.

[CR27] Han X, Chen M, Wang F (2013). Forebrain engraftment by human glial progenitor cells enhances synaptic plasticity and learning in adult mice. Cell Stem Cell.

[CR28] Hostiuc S, Rusu MC, Negoi I (2019). The moral status of cerebral organoids. Regenerative Therapy.

[CR29] Jaworska, A.., and J. Tannenbaum. 2018. The Grounds of Moral Status*, The Stanford Encyclopedia of Philosophy*, ed. E. N. Zalta, https://plato.stanford.edu/archives/spr2018/entries/grounds-moral-status/.

[CR30] Joshi NJ, Tononi G, Koch C (2013). The minimal complexity of adapting agents increases with fitness. PLoS Computational Biology.

[CR31] Kahane G, Savulescu J (2009). Brain damage and the moral significance of consciousness. Journal of Medicine and Philosophy.

[CR32] Kipke R (2019). Being human: Why and in what sense it is morally relevant. Bioethics.

[CR33] Koch C, Massimini M, Boly M, Tononi G (2016). Neural correlates of consciousness: Progress and problems. Nature Reviews Neuroscience.

[CR34] Knoppers BM, Greely HT (2019). Biotechnologies nibbling at the legal “human”. Science.

[CR35] Koplin J, Savulescu J (2019). Time to rethink the law on part-human chimeras. Journal of Law and the Biosciences.

[CR36] Koplin, J., J. Savulescu. 2019b. Moral limits of brain organoid research. *The Journal of Law, Medicine & Ethics*, https://ora.ox.ac.uk/objects/uuid:98bb6cc1-fef4-4022-ae3e-e84d3b1bf9f4. 10.1177/1073110519897789 PMC7433685 31957593

[CR37] Kriegel U (2019). The value of consciousness. Analysis.

[CR38] Lancaster MA, Knoblich JA (2014). Generation of cerebral organoids from human pluripotent stem cells. Nature Protocols.

[CR39] Lancaster MA, Renner M, Martin CA (2013). Cerebral organoids model human brain development and microcephaly. Nature.

[CR88] Lavazza A, Lavazza A (2016). Neuroethics: A New Framework—From Bioethics to Anthropology. Frontiers in Neuroethics.

[CR40] Lavazza A (2019). What (or sometimes who) are organoids? And whose are they?. Journal of Medical Ethics.

[CR41] Lavazza A, Pizzetti FG (2020). Human cerebral organoids as a new legal and ethical challenge. The Journal of Law and the Biosciences.

[CR42] Lavazza A, Massimini M (2018). Cerebral organoids: Ethical issues and consciousness assessment. Journal of Medical Ethics.

[CR43] Lavazza A, Massimini M (2018). Cerebral organoids and consciousness: How far are we willing to go?. Journal of Medical Ethics.

[CR44] Li C, Xu D, Ye Q (2016). Zika virus disrupts neural progenitor development and leads to microcephaly in mice. Cell Stem Cell.

[CR45] MacKenzie, R.J. 2019. Cutting Through the Headlines: Are Scientists Really Growing Sentient "Mini-brains"?, *Technology Networks*, https://www.technologynetworks.com/neuroscience/articles/cutting-through-the-headlines-are-scientists-really-growing-sentient-mini-brains-328000.

[CR46] Mann SP, Sun R, Hermerén G (2019). A framework for the ethical assessment of chimeric animal research involving human neural tissue. BMC Medical Ethics.

[CR47] Mansour AA, Gonçalves JT, Bloyd CW (2018). An in vivo model of functional and vascularized human brain organoids. Nature Biotechnology.

[CR48] Marquis D (1989). Why abortion is immoral. The Journal of Philosophy.

[CR49] Massimini M, Boly M, Casali A (2009). A perturbational approach for evaluating the brain's capacity for consciousness. Progress in Brain Research.

[CR50] Marton RM, Paşca SP (2019). Organoid and assembloid technologies for investigating cellular crosstalk in human brain development and disease. Trends in Cell Biology.

[CR51] Munsie M, Hyun I, Sugarman J (2017). Ethical issues in human organoid and gastruloid research. Development.

[CR52] Nagy A, Rossant J (2002). Chimaeras and mosaics for dissecting complex mutant phenotypes. International Journal of Developmental Biology.

[CR53] Nir Y, Tononi G (2010). Dreaming and the brain: from phenomenology to neurophysiology. Trends in Cognitive Science.

[CR54] Northoff G, Huang Z (2017). How do the brain’s time and space mediate consciousness and its different dimensions? Temporo-spatial theory of consciousness (TTC). Neuroscience and Biobehavioral Reviews.

[CR55] Oizumi M, Albantakis L, Tononi G (2014). From the phenomenology to the mechanisms of consciousness: Integrated information theory 3.0. PLOS Computational Biology.

[CR56] Owen A (2017). Into the Gray Zone: A Neuroscientist Explores the Border Between Life and Death.

[CR57] Pacitti D, Privolizzi R, Bax BE (2019). Organs to cells and cells to organoids: The evolution of in vitro central nervous system modelling. Frontiers in Cellular Neuroscience.

[CR58] Paşca SP (2018). The rise of three-dimensional human brain cultures. Nature.

[CR59] Paşca AM, Sloan SA, Clarke LE (2015). Functional cortical neurons and astrocytes from human pluripotent stem cells in 3D culture. Nature Methods.

[CR60] Pera MF, de Wert G, Dondorp W (2015). What if stem cells turn into embryos in a dish?. Nature Methods.

[CR61] Putnam H (1981). Brains in a vat. In Id., Reason Truth and History.

[CR62] Qian X, Nguyen HN, Song MM (2016). Brain-region-specific organoids using mini-bioreactors for modeling ZIKV exposure. Cell.

[CR90] Qian X, Nguyen HN, Jacob F (2017). Using brain organoids to understand Zika virus-induced microcephaly. Development.

[CR63] Quadrato G, Nguyen T, Macosko EZ (2017). Cell diversity and network dynamics in photosensitive human brain organoids. Nature.

[CR64] Reardon, S. 2019. ‘Outlandish’ competition seeks the brain’s source of consciousness. *Science*, https://www.sciencemag.org/news/2019/10/outlandish-competition-seeks-brain-s-source-consciousness. 10.1126/science.366.6463.293 31624192

[CR65] Rygaard J, Poulsen CO (1969). Heterotransplantation of a human malignant tumour to “Nude” mice. Acta Pathologica Microbiologica Scandinavica.

[CR66] Sakaguchi H, Ozaki Y, Ashida T (2019). Self-organized synchronous calcium transients in a cultured human neural network derived from cerebral organoids. Stem Cell Reports.

[CR67] Sample, I. 2019. Scientists 'may have crossed the ethical line' in growing human brains, *The Guardian*, https://www.theguardian.com/science/2019/oct/21/scientists-may-have-crossed-ethical-line-in-growing-human-brains.

[CR68] Sarasso S, Rosanova M, Casali AG (2014). Quantifying cortical EEG responses to TMS in (Un)consciousness. Clinical EEG and Neuroscience.

[CR69] Sato T, Vries RG, Snippert HJ (2009). Single Lgr5 stem cells build crypt–villus structures in vitro without a mesenchymal niche. Nature.

[CR70] Sawai T, Sakaguchi H, Thomas E (2019). The ethics of cerebral organoid research: Being conscious of consciousness. Stem Cell Reports.

[CR71] Schwartz MP, Hou Z, Propson NE (2015). Human pluripotent stem cell-derived neural constructs for predicting neural toxicity. *P*. Natl. Acad. Sci. U S A..

[CR72] Searle JR (1992). The Rediscovery of the Mind.

[CR73] Setia H, Moutri AR (2019). Brain organoids as a model system for human neurodevelopment and disease. Seminars in Cell & Developmental Biology.

[CR74] Streiffer R (2007). At the edge of humanity: Human stem cells, chimeras, and moral status. The Journal of Philosophical Research.

[CR75] Sun AX, Yuan Q, Fukuda M (2019). Potassium channel dysfunction in human neuronal models of Angelman syndrome. Science.

[CR76] Thomson JJ (1971). A defense of abortion. Philosophy & Public Affairs.

[CR77] Tononi G (2008). Consciousness as integrated information: A provisional manifesto. Biological Bulletin.

[CR78] Tononi G (2012). Integrated information theory of consciousness: An updated account. Archives Italiennes de Biologie.

[CR80] Tononi G, Koch C (2008). The neural correlates of consciousness. Annals of the New York Academy of Sciences.

[CR81] Tononi G, Koch C (2015). Consciousness: here, there and everywhere?. Philosophical Transactions of the Royal Society B.

[CR79] Tononi G, Boly M, Massimini M (2016). Integrated information theory: From consciousness to its physical substrate. Nature Reviews Neuroscience.

[CR82] Tononi G, Sporns O (2003). Measuring information integration. BMC Neuroscience.

[CR83] Trujillo CA, Gao R, Negraes PD (2019). Complex oscillatory waves emerging from cortical organoids model early human brain network development. Cell Stem Cell.

[CR84] Van Gulick, R. 2018. Consciousness, *The Stanford Encyclopedia of Philosophy*, E. N. Zalta (ed.), https://plato.stanford.edu/archives/spr2018/entries/consciousness/.

[CR85] Velasco S, Kedaigle AJ, Simmons SK (2019). Individual brain organoids reproducibly form cell diversity of the human cerebral cortex. Nature.

[CR86] Yin X, Mead BE, Safaee H (2016). Engineering stem cell organoids. Cell Stem Cell.

[CR87] Xiang Y, Tanaka Y, Cakir B (2019). hESC-derived thalamic organoids form reciprocal projections when fused with cortical organoids. Cell Stem Cell.

